# Young Adults' Social Relationships Affect Their Likelihood of Ruminating About Past School‐Age Victimization

**DOI:** 10.1002/ab.70050

**Published:** 2025-09-21

**Authors:** Sarah T. Malamut, Christina Salmivalli

**Affiliations:** ^1^ Department of Psychology INVEST Research Flagship University of Turku Turku Finland

**Keywords:** loneliness, romantic relationships, rumination, victimization, workplace victimization

## Abstract

Rumination about past victimization as an adult underlies the link between school‐age victimization and mental health difficulties in young adulthood. Yet, there is a lack of knowledge regarding the risk factors for adults to ruminate on their past victimization experiences. The current study fills this gap by examining whether current social relationships (e.g., workplace victimization, loneliness, romantic relationship satisfaction) of young adults play a role in rumination (as an adult) on past victimization. This preregistered study uses longitudinal data from 1772 Finnish individuals (*M*_age_ = 26.04, SD = 1.57), who were part of a large longitudinal project when they were in Grades 4–9, with a follow‐up study conducted over a decade later. Workplace victimization and loneliness in adulthood emerged as key predictors of rumination in adulthood about past victimization. The findings suggest that current adult social relationships are a risk factor for previously victimized individuals to dwell on their victimization as adults, regardless of the extent to which they were victimized in adolescence.

## 1 Introduction

Peer victimization (i.e., being the target of aggressive behaviors from peers) is a painful experience for youth, and its ill effects can last into adulthood (McDougall and Vaillancourt 2015). Growing evidence suggests that rumination may be an underlying mechanism by which victimization can lead to negative outcomes (Huang et al. 2023; Labella et al. 2024; Malamut and Salmivalli 2021, 2023; Peets et al. 2022). In addition to being more likely to ruminate in general (e.g., Feinstein et al. 2014), victimized youth may also ruminate specifically on social stressors (Monti et al. 2017). A recent study found an indirect effect of victimization in adolescence on adult depression and aggression via rumination about past victimization (Malamut and Salmivalli 2023). Although rumination about past victimization appears to play an important role in the long‐term adjustment of victimized youth, it is not yet clear under which circumstances young adults are more likely to ruminate about their past experiences as targets of aggression. The current study examines whether the current social relationships of young adults affect the link between adolescent victimization and rumination (as an adult) on past victimization. By focusing on a sample of individuals who had previously been victimized during their school years, the current study will first establish whether young adults’ current struggles in their social relationships are risk factors for rumination on past victimization (regardless of the extent to which they had been victimized during school). The current study will then test whether current social relationships moderate the association between adolescent victimization and rumination (as an adult) on past victimization.

### 1.1 Rumination About Past Victimization

Interpersonal stress has been highlighted as a risk factor for elevated (general) rumination (Watkins and Roberts 2020). Peer victimization is a salient interpersonal stressor that impacts youth, and, as such, may be a risk factor for ruminative tendencies. Indeed, a recent cross‐national study—using retrospective reports of childhood victimization—found support for indirect effects of childhood victimization on depressive symptoms in young adulthood via general ruminative tendencies (Labella et al. 2024). Whereas general ruminative tendencies can arise from stressors, peer victimization has been conceptualized as a form of interpersonal trauma (Idsoe et al. 2021) that can result in stress symptoms such as intrusive thoughts about the victimization itself (Jenkins et al. 2023). Consistent with conceptualizations of intrusive rumination (e.g., Cann et al. 2011), highly stressful events (such as victimization during school years) may result in involuntary, undesired focus on the event—even after time has passed. Thus, it is critical for research to consider not just the general tendency to ruminate, but also the extent to which individuals specifically ruminate on past victimization experiences—as victimization is a stressful event that may result in lasting, stressor‐specific intrusive rumination. In addition, rumination specifically about past victimization has been found to underlie the link between victimization in childhood and both internalizing (Peets et al. 2022) and externalizing problems (Malamut and Salmivalli 2021) in adolescence. This can also extend to longer‐term dynamics—using the same sample of young adults as in the current study, we found that higher levels of adolescent victimization was (modestly) positively associated with rumination about past victimization as an adult (Malamut and Salmivalli 2023). However, it is still unclear which factors are related to whether or not individuals ruminate about past victimization.

This is concerning as rumination is a transdiagnostic vulnerability for a multitude of mental health difficulties (e.g., depression, anxiety; Watkins and Roberts 2020). Still, there is a large degree of heterogeneity amongst victimized youth—not everyone who has been victimized during their school years will dwell on their experiences as adults. Understanding factors that influence whether individuals do or do not ruminate on their past victimization is crucial, given the serious, potentially long‐lasting, consequences that can arise from victimization.

### 1.2 Social Relationships in Adolescence and Adulthood

In adolescence, social interactions and relationships with peers become increasingly important (LaFontana and Cillessen 2010). Indeed, part of why peer victimization is so distressing for youth may be because it can signal a lack of successful interpersonal relationships. Whereas social difficulties (such as victimization) may be transient for some individuals (Oncioiu et al. 2023), other individuals may continue to struggle with their social relationships even into adulthood (e.g., Brendgen and Poulin 2017). Being victimized in adolescence may lead individuals to develop negative relational schemas (Baldwin 1992), which can then hinder social‐cognitive and socioemotional processing (Rosen et al. 2007). When those who have previously been victimized become adults and still struggle socially, these feelings and experiences may reactivate a (negative) relational schema, and lead to repetitive negative thinking about their earlier social struggles in adolescence (e.g., Stavropoulos et al. 2020). In addition, consistent with both the main effects and buffering models of social support (Cohen and Wills 1985), (successful) social relationships in adulthood may both positively contribute to current adjustment (e.g., less rumination) as well as offset the negative consequences of a specific stressful event (such as past experiences of victimization) that is still resulting in rumination.

In the current study, we focus on three different domains of adults’ relationships: workplace victimization, loneliness (i.e., a lack of intimate or supportive relationships), and romantic relationship quality. There is some evidence that individuals who were victimized in their school years are more likely to be the targets of workplace victimization as adults (e.g., Andersen et al. 2015; Brendgen and Poulin 2017; Smith et al. 2003). For individuals who were both victimized in their youth and are still currently victimized as an adult, it may be more difficult to avoid dwelling on their past experiences. The types of mistreatment that they are currently experiencing could trigger the memories of the similar mistreatment they faced during their school years (Ehlers and Clark 2000; Fenneman et al. 2025; Miller et al. 2003). Individuals who remain targeted as adults may also struggle with self‐blame (e.g., there must be something wrong with me because I am still being victimized). Such self‐critical cognitions are also strongly associated with rumination (e.g., Spasojević and Alloy 2001). We expect a similar effect for loneliness, which can include feelings of isolation and rejection (Van Tilburg and De Jong Gierveld 2023)—feelings that are also often part of peer victimization experiences. Therefore, loneliness in adulthood may also be indicative to individuals that they are still struggling socially, and have not successfully established close ties. Similar to workplace victimization, loneliness in adulthood likely serves as a reminder of experiences that they had in adolescence (e.g., reminiscent of feeling victimized or rejected during their school years). Thus, we expect that workplace victimization and loneliness will exacerbate the association between past victimization experiences and rumination about these experiences.

Another (especially salient) form of interpersonal relationships in adulthood is romantic relationships. Although romantic relationships begin to increase in prevalence already in (late) adolescence, forming such relationships is considered a key developmental task in adulthood (Meier and Allen 2009). Given that individuals have a fundamental need for social relationships (Baumeister and Leary 1995), individuals who have established satisfying romantic relationships in adulthood may be less likely to continue to ruminate on past victimization experiences during their school years. In addition, having a successful social relationship such as a romantic relationship could disrupt negative relational schemas that had been formed in adolescence. Thus, we expected romantic relationship satisfaction to weaken the association between victimization during school years and rumination about past victimization as an adult.

## 2 Methods

### 2.1 Participants and Procedure

Participants were Finnish individuals who were part of a large longitudinal project when they were in Grades 4‐9 (*n* = 22,135; see Kärnä et al. 2011, 2013), with a follow‐up study conducted 13–15 years later when they were young adults. Data collection for the follow‐up study concluded in May 2023, with a total of 4176 participants (18.9% of the original sample; *M*_age_ = 12.84, SD = 2.13 in the initial study). Participants in the follow‐up study had lower levels of peer‐reported victimization (*M* = 0.06, SD = 0.07) than those who did not participate (*M* = 0.059, SD = 0.08) (*t* = 3.50, *p* < 0.001). However, there was no significant difference in self‐reported victimization between those who participated in the follow‐up study (*M* = 0.48, SD = 0.79) and those who did not participate (*M* = 0.50, SD = 0.81) (*t* = 1.67, *p* = 0.095). There was also no significant difference in depressive symptoms in participants in the follow‐up study (*M* = 0.73, SD = 0.67) versus those who did not participate (*M* = 0.71, SD = 0.68) (*t* = −1.64, *p* = 0.101).

Only a subsample of the 4176 participants who had self‐reported being victimized “at least once” during school as adolescents (see Measures) were included in this study, given our focus on rumination about past victimization (*n* = 1772; 59.7% self‐identified as women; *M*_age_ = 26.04, SD = 1.57; 88.1% born in Finland and 82.7% with parents born in Finland) in the follow‐up study. Across variables, missingness ranged from 0 (self‐reported victimization) to 33% (romantic relationship satisfaction). The data were not MCAR (Little 1988), χ^2^ = 267.58, *df* = 84, *p* < 0.001.

In the initial study, adolescents completed online questionnaires during school at three time points across two school years (May, December, May). Teachers supervised the administration of the questionnaires with detailed instructions. The students were assured that their answers were confidential, and informed that they could stop participating at any time. For the follow‐up study, research assistants mailed letters to the original participants with details of the new data collection and a link to access the online questionnaire. This procedure was in concordance with the ethical principles of Finnish National Board on Research Integrity (TENK) and the BLINDED Ethics Committee for Human Sciences. This study was preregistered (https://osf.io/ugvtc/?view_only=e59053f285fa41b4b9cdc4ef2cd817f6).

### 2.2 Measures

#### 2.2.1 Victimization (Adolescence)

Victimization in adolescence was assessed using the global item from the Olweus Bully/Victim questionnaire (OBVQ‐R; Olweus 1996) during waves 1–3. Students were provided the Olweus (1996) definition of bullying (highlighting the intentional, harmful, and repetitive nature of bullying), then asked “How often have you been bullied by others at school in the last 2 months?” Participants responded on a five‐point scale (0 = ‘*not at all*,’ 4 = ‘*several times a week*’). Participants responses at waves 1–3 were averaged to form one score representing adolescent self‐reported victimization.

Peer‐reported victimization was also assessed in adolescence, using three items from the Participant Role Questionnaire (PRQ: (Salmivalli and Voeten 2004); e.g., ‘s/he is called names and made fun of’). Participants could nominate an unlimited number of classmates for each item. The majority of classrooms (99%) had a participation rate of at least 40% at each wave. The received nominations were summed and divided by the number of possible nominators within each classroom to form a proportion score. A peer‐reported victimization score was calculated at each wave by averaging the proportion scores across the three PRQ items. The three scores were then averaged to form one score representing adolescent peer‐reported victimization.

#### 2.2.2 Workplace Victimization (Adulthood)

Workplace victimization in adulthood was assessed using the 20‐item Aggressive Experiences Scale (AES: Glomb and Liao 2003) focusing on being the target of aggression in the workplace. Participants were asked how often they were the target of different hostile acts at work (e.g., ‘belittling your opinions in front of others’) from their supervisor or coworkers on a 5‐point Likert scale (0 = ‘*Never*,’ 4 = ‘*Once a week or more*’). A total score was then computed by averaging the 20 items (*α* = 0.92).

#### 2.2.3 Loneliness (Adulthood)

Loneliness in adulthood was assessed using the 11‐item de Jong Gierveld Loneliness scale (De Jong‐Gierveld and Kamphuls 1985). Participants were instructed to answer each item (e.g., ‘There is always someone I can talk to about my daily problems’) in regard to their current feelings. Participants responded on a 5‐point Likert scale (1 = ‘*yes!*’, 5 = ‘*no!*’), where higher scores indicate more loneliness (with six items reverse‐coded). A total score was formed by averaging across the 11 items (*α* = 0.90).

#### 2.2.4 Romantic Relationship Satisfaction (Adulthood)

Romantic relationship satisfaction in adulthood was measured using the 7‐item Relationship Assessment Scale (Hendrick 1988). Participants responded to the items (e.g., ‘In general, how satisfied are you with your relationship?’) on a 3‐point Likert scale (1 = ‘*unsatisfied*,’ 3 = ‘*extremely satisfied*’). Two items were reverse‐coded, such that higher scores indicate higher romantic relationship quality. A total score was formed by averaging across the 7 items (*α* = 0.85).

#### 2.2.5 Rumination about Past Victimization (Adulthood)

Participants were asked “how often were you bullied at school?”, on a 4‐point Likert scale (1 = ‘*Never*,’ 4 = ‘*Repeatedly, every week*’). Only participants who reported being bullied at least once were asked to answer eight items about rumination on past victimization (e.g., ‘I can't stop thinking about what the bullies did to me’) on a 4‐point Likert scale (1 = ‘*Not true at all,*’ 4 = ‘*Completely true*’) (*α* = 0.96). This measure was based on a rumination scale about transgressions (McCullough et al. 2007), adapted to victimization (Peets et al. 2022). Responses were recoded as “0” (value of 1—“not true at all”) or “1” (values of 1 or more on the scale). Of the 1772 participants in this study (who all had self‐reported being victimized in adolescence), 208 did not *retrospectively* report having been bullied during their school years. These individuals were given a “0” on the rumination about past victimization measure, due to not remembering or endorsing having been victimized during their school years (i.e., no rumination about past victimization). An exploratory descriptive analysis found that the 208 individuals who did not retrospectively report having been bullied also had: lower levels of self‐reported adolescent victimization (*M* = 0.85, SD = 0.73 vs. *M* = 1.16, SD = 0.88, *p* < 0.001), lower peer‐reported levels of adolescent victimization (*M* = 0.04, SD = 0.04 vs. *M* = 0.09, SD = 0.09, *p* < 0.001), and lower levels of adolescent depressive symptoms (*M* = 0.76, SD = 0.63 vs. *M* = 0.93, SD = 0.76, *p* < 0.001) compared to the rest of the sample.

### 2.3 Control Variables

#### 2.3.1 Depressive Symptoms (Adolescence)

In adolescence, participants answered a 7‐item scale derived from the Beck Depression Inventory (BDI; Beck et al. 1996; Williford et al. 2012) focusing on cognitive‐affective concerns (e.g., ‘What is your mood like?’) on a 5‐point Likert scale (e.g., 0 = ‘*Fairly bright and good*,’ 4 = ‘*I am so depressed and downcast that I cannot take it anymore*’). Higher scores reflect greater depressive symptoms (*αs* = 0.88, 0.92, 0.93 at waves 1–3, respectively). A total score for depressive symptoms in adolescence was calculated by averaging across the 3 waves (*rs* > 0.49).

#### 2.3.2 Age and Gender

We controlled for age and gender. For gender, two dummy‐coded variables were created, given that 2.1% of participants chose not to answer or responded “other.” Women were the reference group, with a dummy‐coded variable for men (1 = *men*, 0 = *women/other*) and for participants who chose not to answer or responded “other” (1 = *other*, 0 = *men/women*).

## 3 Analytic Plan

We conducted two sets of analyses in R. The first included a series of logistic regressions, predicting whether or not individuals engaged in *any* rumination about past victimization (0 = *no rumination*, 1 = *some rumination*). For the logistic regressions, missing data was handled using multiple imputation (with 50 imputations), using the mice package (van Buuren and Groothuis‐Oudshoorn 2011) in R. The second analyses included a series of linear regressions, predicting the *extent* to which individuals ruminated about past victimization. For the linear regressions, missing data was handled using full information maximum likelihood (FIML) with lavaan (Rosseel 2012) in R. Age and gender of the participants were controlled for in all models. We also controlled for depressive symptoms in adolescence, as this could impact individuals’ tendency to ruminate. Three possible indicators of adult social relationships (workplace victimization, loneliness, and romantic relationship satisfaction) were tested in separate models. For each model, we first included the main effects of each variable, then tested them as moderators by adding a two way interaction between adolescent victimization and the adult social relationship indicator. All continuous variables were grand‐mean centered. As all variables were assessed with self‐reports, and thus at risk for shared method bias, we also tested models in which peer‐reported victimization was the predictor rather than self‐reported victimization.

## 4 Results

Descriptive statistics and correlations are presented in Table 1. Among this sample of individuals who were victimized at least once during adolescence, self‐reported victimization in adolescence was positively associated with peer‐reported victimization (*r* = 0.36) and depressive symptoms (*r* = 0.22) in adolescence. Both self‐ and peer‐reported victimization in adolescence were positively associated with loneliness (*rs* = 0.07, 0.08, respectively) and rumination about past victimization (*rs* = 0.12, 0.17, respectively) in adulthood. Self‐reported victimization was also positively associated with workplace victimization (*r* = 0.07) in adulthood.

**Table 1:** Descriptive statistics and correlations of study variables.

	1	2	3	4	5	6	*M* (SD)
*Adolescence*							
1. Self‐reported victimization	—						1.12 (0.87)
2. Peer‐reported victimization	0.36***	—					0.08 (0.09)
3. Depressive symptoms	0.22***	0.07**	—				0.91 (0.75)
*Young Adulthood*							
4. Workplace victimization	0.09***	0.03	0.14***	—			1.35 (0.49)
5. Loneliness	0.07**	0.08**	0.20***	0.19***	—		2.51 (0.79)
6. Romantic relationship satisfaction	−0.02	−0.05	−0.12***	−0.16***	−0.30***		2.71 (0.34)
7. Rumination about past victimization	0.12***	0.17***	0.20***	0.20***	0.32***	−0.11***	1.73 (0.76)

** *p* < 0.01

*** *p* < 0.001.

### 4.1 Predictors of Rumination About Past Victimization

#### 4.1.1 Logistic Regressions

We conducted a series of logistic regressions to test predictors of whether adults engaged in any rumination about past victimization in their adolescence, controlling for depressive symptoms in adolescence, age and gender. The extent to which they had been victimized in adolescence (self‐reported victimization) was associated with rumination about past victimization—those with higher levels of self‐reported victimization were more likely to ruminate about past victimization. When testing the main effects of individuals’ current social relationships in adulthood (Table 2), those with higher levels of workplace victimization and loneliness were more likely to engage in rumination about past victimization experiences, whereas those with higher levels of romantic relationship satisfaction were less likely. However, there were no significant interactions between self‐reported victimization in adolescence and any social relationship indicator in adulthood (*ps* >= 0.27). The same pattern of main effects were found when including peer‐reported victimization in the models instead of self‐reported victimization (Table 2). There also were no significant interactions between peer‐reported victimization in adolescence and any social relationship indicator in adulthood (*ps* >= 0.61).

**Table 2:** Logistic regressions predicting adult rumination about adolescent victimization experiences.

	Self‐reported victimization			Peer‐reported victimization		
	*b* (SE)	*OR*	95% CI	*b* (SE)	*OR*	95% CI
Intercept	0.69*** (0.01)	—	—	0.69*** (0.01)	—	—
Victimization	0.06*** (0.01)	1.06	(1.03, 1.09)	0.79*** (0.13)	2.19	(1.71, 2.81)
Age	0.03*** (0.01)	1.03	(1.01, 1.04)	0.02** (0.01)	1.02	(1.01, 1.04)
Gender (man)	−0.14*** (0.02)	0.87	(0.83, 0.91)	−0.14*** (0.02)	0.87	(0.83, 0.91)
Gender (other)	0.06 (0.08)	1.07	(0.92, 1.24)	0.04 (0.08)	1.04	(0.89, 1.21)
Depressive symptoms	0.05** (0.02)	1.05	(1.01, 1.08)	0.06*** (0.02)	1.06	(1.03, 1.09)
Workplace victimization	0.10*** (0.02)	1.11	(1.06, 1.16)	0.11*** (0.02)	1.11	(1.06, 1.17)
Intercept	0.69*** (0.01)	—	—	0.69*** (0.01)	—	—
Victimization	0.06*** (0.01)	1.07	(1.04, 1.09)	0.73*** (0.12)	2.08	(1.63, 2.65)
Age	0.03*** (0.01)	1.03	(1.02, 1.05)	0.03** (0.01)	1.03	(1.01, 1.04)
Gender (man)	−0.14*** (0.02)	0.87	(0.83, 0.91)	−0.14*** (0.02)	0.87	(0.83, 0.91)
Gender (other)	0.04 (0.08)	1.04	(0.90, 1.21)	0.02 (0.08)	1.02	(0.88, 1.18)
Depressive symptoms	0.02 (0.02)	1.02	(0.99, 1.06)	0.04* (0.02)	1.04	(1.01, 1.07)
Loneliness	0.14*** (0.01)	1.15	(1.11, 1.18)	0.13*** (0.01)	1.14	(1.11, 1.17)
Intercept	0.69*** (0.01)	—	—	0.69*** (0.01)	—	—
Victimization	0.07*** (0.01)	1.07	(1.04, 1.10)	0.79*** (0.13)	2.20	(1.72, 2.83)
Age	0.03*** (0.01)	1.03	(1.02, 1.04)	0.02** (0.01)	1.02	(1.01, 1.04)
Gender (man)	−0.16*** (0.02)	0.85	(0.81, 0.90)	−0.15*** (0.02)	0.86	(0.82, 0.90)
Gender (other)	0.08 (0.08)	1.08	(0.93, 1.26)	0.05 (0.08)	1.05	(0.90, 1.22)
Depressive symptoms	0.04** (0.02)	1.05	(1.01, 1.08)	0.06*** (0.02)	1.06	(1.03, 1.09)
Romantic relationship satisfaction	−0.13*** (0.04)	0.88	(0.81, 0.94)	−0.12** (0.04)	0.89	(0.82, 0.95)

Abbreviations: 95% CI, 95% confidence intervals for the odds ratios; b, unstandardized coefficients; OR, odds ratio; SE, standard errors.

* *p* < 0.05

** *p* < 0.01

*** *p* < 0.001.

As an exploratory analysis, we included all social relationship indicators in adulthood in the same model. In this model, workplace victimization and loneliness still positively predicted any engagement in rumination about past victimization, but romantic relationship satisifcation was no longer a significant predictor (*p* = 0.31). Again, there were no significant interactions between victimization and social relationships in adulthood, and the same pattern was found when using peer‐reported victimization.

#### 4.1.2 Linear Regressions

**Figure 1:**
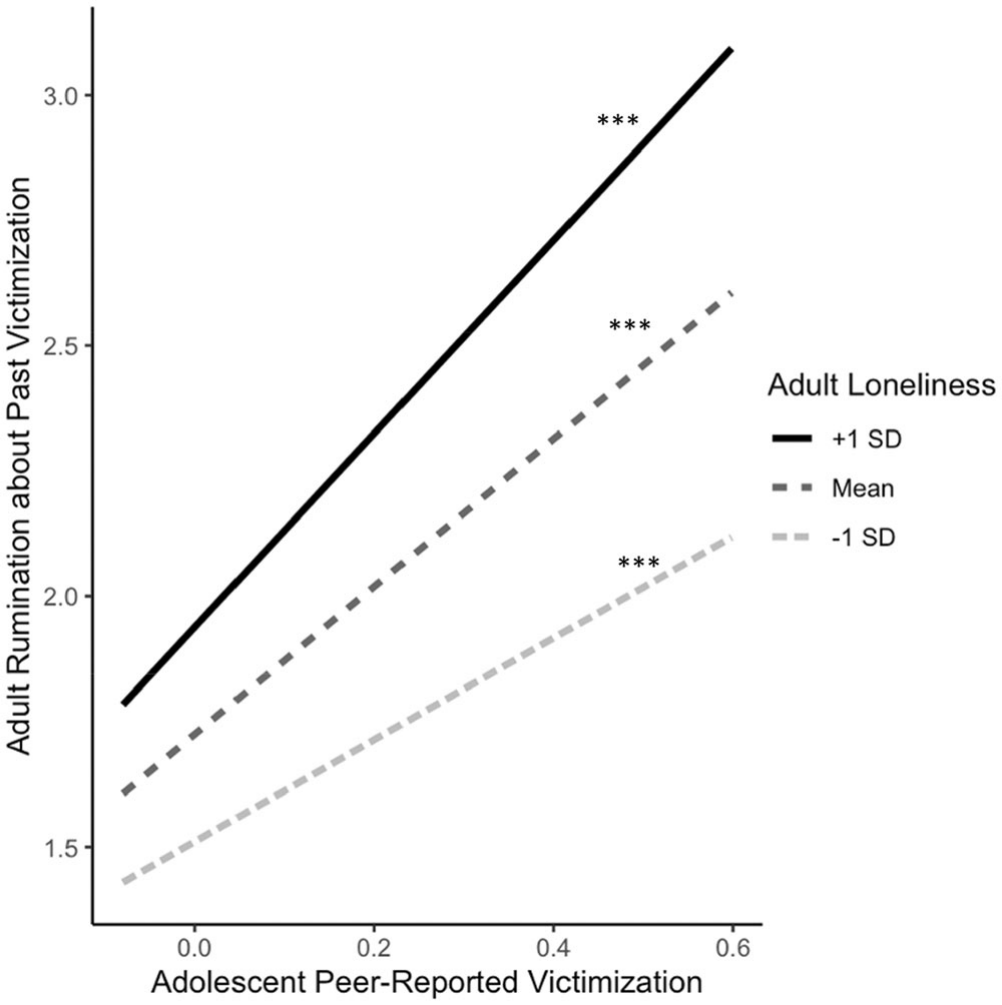
Adult loneliness moderates the association between adolescent peer‐reported victimization and adult rumination about past victimization. ***p < 0.001.

Next, we conducted a series of linear regressions to test predictors of the extent to which individuals ruminated about their adolescent victimization experiences as adults, again controlling for depressive symptoms in adolescence, age and gender. There was a positive association between self‐reported victimization in adolescence and rumination about past victimization as an adult. When testing the main effects of individuals’ current social relationships in adulthood (Table 3), workplace victimization (*b* = 0.25, 95% CI: 0.18, 0.32) and loneliness (*b* = 0.28, 95% CI: 0.24, 0.32) were associated with higher levels of rumination about past victimization experiences, whereas romantic relationship satisfaction (*b* = −0.25, 95% CI: −0.38, −0.13) was negatively associated with rumination about past victimization. There were no significant interactions between self‐reported victimization in adolescence and any social relationship indicator in adulthood (Table 3). When testing peer‐reported victimization, there was a similar pattern of main effects, as well as one significant interaction (Table 3). There was a significant interaction between peer‐reported victimization in adolescence and loneliness in adulthood (*p* = 0.009). Although the slope was positive and significant at low (*b* = 1.01, SE = 0.28, *p* < 0.001), average (*b* = 1.47, SE = 0.19, *p* < 0.001), and high (*b* = 1.93, SE = 0.24, *p* < 0.001) levels of loneliness, it was strongest at high levels of loneliness (see Figure 1). Tests of regions of significance (using the Johnson–Neyman technique) indicated that the slope of peer‐reported victimization was significant when adult loneliness was greater than −1.29, with (centered) observed values in our sample ranging from −1.51 to 2.30.

**Table 3:** Linear regressions predicting adult rumination about adolescent victimization experiences.

	Self‐reported victimization				Peer‐reported victimization			
	Main effects		Interactions		Main effects		Interactions	
	*b* (SE)	*β*	*b* (SE)	*β*	*b* (SE)	*β*	*b* (SE)	*β*
Intercept	1.73*** (0.02)	—	1.73*** (0.02)	—	1.73*** (0.02)	—	1.73*** (0.02)	—
Victimization	0.11*** (0.02)	0.13	0.11*** (0.02)	0.13	1.67*** (0.19)	0.20	1.66** (0.19)	0.20
Age	0.03** (0.01)	0.07	0.03** (0.01)	0.07	0.02* (0.01)	0.05	0.02* (0.01)	0.05
Gender (man)	−0.23*** (0.04)	−0.15	−0.23*** (0.04)	−0.15	−0.24*** (0.04)	−0.15	−0.24*** (0.04)	−0.15
Gender (other)	0.09 (0.12)	0.02	0.09 (0.12)	0.02	0.03 (0.12)	0.01	0.02 (0.12)	0.00
Depressive symptoms	0.12*** (0.02)	0.12	0.12*** (0.02)	0.12	0.14*** (0.02)	0.14	0.14*** (0.02)	0.14
Workplace victimization	0.25*** (0.04)	0.16	0.27*** (0.04)	0.17	0.26*** (0.04)	0.17	0.26*** (0.04)	0.17
Victimization X Workplace Victimization	—	—	−0.07 (0.04)	−0.04	—	—	−0.21 (0.43)	−0.01
Intercept	1.73*** (0.02)	—	1.73*** (0.02)	—	1.73*** (0.02)	—	1.73*** (0.02)	—
Victimization	0.11*** (0.02)	0.13	0.11*** (0.02)	0.13	1.54*** (0.19)	0.19	1.47*** (0.19)	0.18
Age	0.04*** (0.01)	0.09	0.04*** (0.01)	0.09	0.03** (0.01)	0.06	0.03** (0.01)	0.07
Gender (man)	−0.23*** (0.04)	−0.15	−0.23*** (0.04)	−0.15	−0.23*** (0.04)	−0.15	−0.23*** (0.03)	−0.15
Gender (other)	0.05 (0.11)	0.01	0.05 (0.11)	0.01	−0.00 (0.11)	−0.00	−0.01 (0.11)	−0.00
Depressive symptoms	0.08** (0.02)	0.08	0.08** (0.02)	0.08	0.10*** (0.02)	0.10	0.10*** (0.02)	0.10
Loneliness	0.28*** (0.02)	0.30	0.28*** (0.02)	0.30	0.27*** (0.02)	0.28	0.27*** (0.02)	0.28
Victimization X Loneliness	—	—	.00 (0.02)	0.00	—	—	0.59** (0.23)	0.06
Intercept	1.74*** (0.02)	—	1.74*** (0.02)	—	1.74*** (0.02)	—	1.74*** (0.02)	—
Victimization	0.12*** (0.02)	0.14	0.12*** (0.02)	0.14	1.67*** (0.19)	0.20	1.66*** (0.19)	0.20
Age	0.04*** (0.01)	0.08	0.04*** (0.01)	0.08	0.03* (0.01)	0.05	0.02* (0.01)	0.05
Gender (man)	−0.26*** (0.04)	−0.17	−0.27*** (0.04)	−0.17	−0.26*** (0.04)	−0.17	−0.26*** (0.04)	−0.17
Gender (other)	0.11 (0.12)	0.02	0.12 (0.12)	0.02	0.05 (0.12)	0.01	0.05 (0.12)	0.01
Depressive symptoms	0.12*** (0.03)	0.12	0.12*** (0.03)	0.12	0.15*** (0.02)	0.15	0.15*** (0.02)	0.15
Romantic relationship satisfaction	−0.25*** (0.06)	−0.12	−0.25*** (0.06)	−0.12	−0.23*** (0.06)	−0.11	−0.23*** (0.06)	−0.11
Victimization X Romantic relationship satisfaction	—	—	−0.06 (0.07)	−0.02	—	—	−0.93 (0.80)	−0.03

Abbreviations: b, unstandardized coefficients; SE, standard errors; *β*, standardized coefficients.

* *p* < 0.05

** *p* < 0.01

*** *p* < 0.001.

As an exploratory analysis, we included all social relationship indicators in adulthood in the same model. Again, workplace victimization and loneliness were positively associated with rumination about past victimization, but romantic relationship satisfaction was no longer a significant predictor (*p* = 0.29). There were no significant interactions between self‐reported victimization and social relationships in adulthood. When all social relationship indicators (and their respective interactions with victimization) were tested in the same model, the interaction between peer‐reported victimization and loneliness remained significant (*p* = 0.016).

### 4.2 Sensitivity Analyses

The main focus of this study was risk factors for ruminating specifically about past victimization, given that victimization is a serious interpersonal stressor that may lead to lasting intrusive thoughts and repetitive negative thinking as well as subsequent mental health difficulties. However, victimization has been linked to both rumination specifically about past victimization but also general rumiantive tendencies, and it is unclear from the existing literature the extent to which ruminating specifically about past victimization is distinct from more general ruminative tendencies. Thus, as sensitivity analyses, we reran all the models also controlling for general sad rumination, which was moderately correlated with rumination specifically about past victimization (*r* = 0.46, *p* < 0.001). All pattern of findings remained the same, with one exception: romantic satisifaction was no longer a significant predictor in any model (*ps* >= 0.13).

## 5 Discussion

Previous research has identified rumination as a mechanism by which interpersonal stressors—such as peer victimization—negatively impact mental health. Indeed, rumination specifically about past victimization experiences has been found to be one pathway in which victimization during school years is linked to mental health problems even in adulthood. Despite the key role that rumination seems to play in this process, very little is known regarding risk factors for whether or not adults ruminate about their past victimization experiences during their school years. The current study first tested whether individuals who still struggle socially as adults would be more likely to ruminate about their past school‐age victimization exepriences, as well as whether these social relationships moderated the link between victimization frequency and rumination about past victimization.

Among a sample of adults who had been victimized at least once or twice as adolescents, workplace victimization and loneliness positively predicted both whether they ruminated *at all* about past victimization, as well as the *extent* to which they ruminated about their past victimization. In contrast, romantic relationship satisfaction was negatively associated with both the likelihood of ruminating at all about past victimization as well as the degree to which they ruminated. Of the three tested predictors, the findings appear most robust for workplace victimization and loneliness in adulthood, as romantic relationship satisfaction was no longer a significant predictor when testing the social relationship indicators simultaneously. Overall, these findings suggest that individuals who struggle socially in adulthood are more likely to dwell on their past victimization experiences. This then can ultimately put individuals at increased risk for mental health problems, given previous findings of the indirect path between peer victimization and mental health via rumination (e.g., Malamut and Salmivalli 2023). Consistent with the idea that not all victimized youth will go on to rumination about their past victimization, we also found a subsample of individuals who had been victimized at least once or twice as adolescents, but who as adults retrospectively reported never having been victimized (reflecting no rumination on the school‐aged experiences). As supported by exploratory descriptive analyses, these individuals did not experience as severe of victimization during their school years. Thus, it is possible that these experiences were not as memorable to these individuals as adults, or did not reach a threshold where as adults they consider that themselves having experienced bullying.

We also examined whether workplace victimization, loneliness, or romantic relationship satisfaction in adulthood moderated the link between victimization in adolescence and rumination in adulthood about past victimization. In most models, there were no significant interactions between victimization in adolescence and social relationships in adulthood. Of note, we found a similar pattern with self‐ and peer‐reported victimization, suggesting that the findings are not primarily driven by shared method bias. Taken together, this suggests that current social relationships struggles in adulthood are a risk factor for previously victimized individuals to dwell on their victimization as adults, regardless of the extent to which they were victimized in adolescence. There was, however, one exception—a significant interaction between peer‐reported victimization and loneliness. In general, higher levels of peer‐reported victimization was associated with higher levels of rumination about past victimization; however, this link was strongest for those who were highest in loneliness. We did not find a similar interaction between self‐reported victimization and loneliness, which could be a reflection of the informant of victimization. That is, individuals who were seen as highly victimized according to peer reports may represent youth who very visibly struggled socially or were seen as outcasts. For these individuals, loneliness in adulthood may be particularly triggering as it is reminiscent of their isolation in adolescence.

The findings of the current study tap into a potential negative cycle for victimized youth, as social success in adolescence (e.g., being a desirable friend) is linked to stronger adult friendships (Allen et al. 2014). Thus, those who were victimized in adolescence may be less likely to establish successful social relationships in adulthood (D'Urso et al. 2023), which then contributes to individuals being more likely to dwell on past victimization experiences.

### 5.1 Strengths, Limitations and Future Directions

This preregistered study includes a large sample of individuals who had previously been victimized. In addition, we utilized a prospective, longitudinal design, whereas many studies examining the impact of school‐age victimization on adult adjustment have relied on retrospective reports of victimization (e.g., Labella et al. 2024). However, there were also a number of limitations. First, key study variables were assessed with self‐reports, which could be susceptible to shared method bias. To address this, we also conducted our analyses using peer‐reported victimization as a predictor rather than self‐reported victimization.

Second, the variables assessed in adulthood (rumination about past victimization, workplace victimization, loneliness, and romantic relationship satisfaction) were all measured at the same time point. Although in the current study we were interested in the adult social relationship indicators as predictors of rumination about past victimization, future research could examine possible bidirections of these associations. For example, it is conceivable that individuals who are prone to dwelling on their past victimization also then are more likely to struggle socially over time (e.g., in a process analogous to interpersonal theories of depression; Hames et al. 2013).

Third, although victimization in adolescence, even when controlling for adolescent depressive symptoms, was a significant predictor of adult rumination about past victimization, it is important to note that the effect sizes were relatively small. This is not surprising, given that the follow‐up study took place over a decade after the original study. Still, this study adds to the evidence of the potentially long‐lasting negative consequences of victimization, and elucidates risk factors for continuing to dwell on victimization experiences even over a decade later.

Lastly, although victimization in adolescence was measured across 3 waves, these waves only spanned two academic years and thus still only offer a snapshot of a relatively short period of time during adolescence. It is possible that some individuals who were not included in our final sample had been victimized at a different age that we could not account for.

## 6 Conclusion

Although rumination—and rumination about past victimization specifically—has been found to play a key role in the lasting negative effects of victimization during school on mental health (Malamut and Salmivalli 2023), less is known regarding the risk factors of ruminating on past victimization. The current study fills this gap by demonstrating that lack of successful social relationships in adulthood is a risk factor for adults to ruminate about past school‐age victimization. Conversely, our findings suggest that helping young adults develop healthy relationships and connect with others may help avoid lasting negative effects of school‐age victimization.

## Data Availability

The data that support the findings of this study are available from the corresponding author upon reasonable request.
